# Physical Activity Monitoring Using a Fitbit Device in Ischemic Stroke Patients: Prospective Cohort Feasibility Study

**DOI:** 10.2196/14494

**Published:** 2021-01-19

**Authors:** Irene Katzan, Andrew Schuster, Tyler Kinzy

**Affiliations:** 1 Neurological Institute Center for Outcomes Research and Evaluation Cleveland Clinic Cleveland, OH United States; 2 Cerebrovascular Center Cleveland Clinic Cleveland, OH United States

**Keywords:** physical activity, accelerometer, ischemic stroke, step activity monitor

## Abstract

**Background:**

Continuous tracking of ambulatory activity in real-world settings using step activity monitors has many potential uses. However, feasibility, accuracy, and correlation with performance measures in stroke patients have not been well-established.

**Objective:**

The primary study objective was to determine adherence with wearing a consumer-grade step activity monitor, the Fitbit Charge HR, in home-going ischemic stroke patients during the first 90 days after hospital discharge. Secondary objectives were to (1) determine accuracy of step counts of the Fitbit Charge HR compared with a manual tally; (2) calculate correlations between the Fitbit step counts and the mobility performance scores at discharge and 30 days after stroke; (3) determine variability and change in weekly step counts over 90 days; and (4) evaluate patient experience with using the Fitbit Charge HR poststroke.

**Methods:**

A total of 15 participants with recent mild ischemic stroke wore a Fitbit Charge HR for 90 days after discharge and completed 3 mobility performance tests from the National Institutes of Health Toolbox at discharge and Day 30: (1) Standing Balance Test, (2) 2-Minute Walk Endurance Test, and (3) 4-Meter Walk Gait Speed Test. Accuracy of step activity monitors was assessed by calculating differences in steps recorded on the step activity monitor and a manual tally during 2-minute walk tests.

**Results:**

Participants had a mean age of 54 years and a median modified Rankin scale score of 1. Mean daily adherence with step activity monitor use was 83.6%. Mean daily step count in the first week after discharge was 4376. Daily step counts increased slightly during the first 30 days after discharge (average increase of 52.5 steps/day; 95% CI 32.2-71.8) and remained stable during the 30-90 day period after discharge. Mean step count difference between step activity monitor and manual tally was –4.8 steps (–1.8%). Intraclass correlation coefficients for step counts and 2-minute walk, standing balance, and 4-meter gait speed at discharge were 0.41 (95% CI –0.14 to 0.75), –0.12 (95% CI –0.67 to 0.64), and 0.17 (95% CI –0.46 to 0.66), respectively. Values were similarly poor at 30 days.

**Conclusions:**

The use of consumer-grade Fitbit Charge HR in patients with recent mild stroke is feasible with reasonable adherence and accuracy. There was poor correlation between step counts and gait speed, balance, and endurance. Further research is needed to evaluate the association between step counts and other outcomes relevant to patients, including patient-reported outcomes and measures of physical function.

## Introduction

Stroke is a leading cause of long-term disability in adults [1]. Ambulation difficulties contribute substantially to long-term disability and health care utilization poststroke [2]. Because of the importance of the ability to ambulate to perform routine activities, ambulatory ability is frequently included as part of the assessment of physical functioning of patients with stroke [3]. In addition, ambulation is recommended for stroke survivors because it has a wide range of benefits that support recovery and cardiovascular health [4].

The ability to passively capture and track continuous ambulatory activity over time in real-world settings for patients with stroke has many potential uses. For example, such tracking may be able to detect relevant changes in a patient’s ability to ambulate more accurately and efficiently than other common strategies, which include self-reported ambulation questionnaires or mobility performance test [5,6]. Self-reported ambulation questionnaires have poor accuracy [7-9]. Performance tests such as gait speed require measurement by a trained assessor, typically in a clinical setting, and they are resource intensive and not always feasible [5]. Another potential use of tracking ambulatory activity in real-time is to provide greater insight into the trajectories of an individual’s recovery after stroke and the effectiveness of new therapies. The use of clinical information derived from real-world settings has been advocated as an avenue for comparative effectiveness research in the field of stroke and other conditions [10]. In addition, passive tracking of ambulatory activity may be able to identify slower recovery patterns of stroke patients who would benefit from targeted interventions; this use has been previously postulated in patients recovering from orthopedic surgery [11,12]. Further, monitoring ambulatory activity can also be used to encourage greater physical activity and physical fitness, which has previously been demonstrated to be effective in studies of people with diabetes [13], and in patients who have recently undergone knee and hip arthroplasty [14].

Step counts can be monitored using step activity monitors. The devices developed for research settings have been expensive and difficult to use [15]. Consumer-oriented step activity monitors are being used more broadly, as they have become more robust and comfortable to wear, with more available features. One in six (15%) consumers in the US currently use health care wearables, including smart-watches or fitness bands [16]. Earlier versions of consumer-oriented step activity monitors were pedometers consisting of mechanical sensors or uniaxial accelerometers, which measure acceleration. These had lower step count accuracy than research-grade devices; many that are currently available now contain more accurate triaxial accelerometers [17,18]. Step counts of newer step activity monitors, including several tested Fitbit devices, have excellent correlation with research-grade accelerometers [15,19,20].

Despite the theoretical potential for the use of easy-to-wear step activity monitors to improve the clinical care and outcomes of patients poststroke, the feasibility and utility of using consumer-oriented step activity monitors in stroke patients are poorly known. The consumer-oriented step activity monitors, Fitbit Charge HR and Garmin Vivosmart, were found to accurately measure step count in 37 patients attending either inpatient or outpatient therapy for stroke who wore the step activity monitors 5 to 10 hours per day for 2 days [15]. In addition to accuracy, other central aspects of the feasibility of using a step activity monitor as part of research or clinical care include adherence, patient experience, and the ability of step counts to serve as a correlate of other outcome measures [21]. Understanding the fluctuations in step counts, specifically in patients with stroke who may have different ambulatory patterns compared to other patient groups, will also be helpful to assess the feasibility of identifying changes or trends in ambulatory activity.

Therefore, we performed a prospective cohort pilot study to assess feasibility of monitoring step counts in ambulatory patients with recent mild ischemic stroke using a consumer step activity monitor, the Fitbit Charge HR. The primary study objective was to determine adherence with wearing a Fitbit Charge HR in home-going ischemic stroke patients during the first 90 days after hospital discharge. Secondary objectives were to (1) determine accuracy of step counts of the Fitbit Charge HR compared with a manual tally; (2) calculate correlations between the Fitbit step counts and the mobility performance scores at discharge and 30 days after stroke; (3) determine variability and change in weekly step counts over 90 days; and (4) evaluate patient experience with using the Fitbit Charge HR poststroke.

## Methods

### Procedures

#### Overview

All admissions to the stroke service were monitored by a trained research coordinator to identify potentially eligible patients. Prior to hospital discharge, participants were given a Fitbit Charge HR step activity monitor and instructed to wear this continuously throughout the day for 90 days. Participants were asked to complete a diary of their physical activity for the first 7 days postdischarge (See Multimedia Appendix 1: Participant Activity Diary). No recommendations were given on amount of physical activity and there were no daily step targets. Participants underwent mobility performance testing of balance, walking endurance, and gait speed shortly before hospital discharge and then again at 30 days postdischarge. They were reimbursed $30 for their time completing the performance testing at the follow-up visit, provided a $10 parking voucher, and were allowed to keep the Fitbit Charge HR for their own personal use at the end of their study participation. Participants were contacted by phone on Day 14 and Day 90 (study end) to be asked about issues with the step activity monitor and the presence of any adverse events stemming from its use. They were then sent a voluntary participant experience survey at the conclusion of the study and asked to mail back the completed survey.

Informed consent was obtained from all patient participants. The study was approved by the Cleveland Clinic Institutional Review Board.

#### Participants

Participants were recruited through daily screening of the inpatient stroke service at Cleveland Clinic. All patients who met eligibility criteria were approached for consent. Initial inclusion criteria were the following: (1) admission to the Stroke Service with an admission diagnosis of ischemic stroke; (2) reside within Cuyahoga County or 5 surrounding counties; (3) informed consent obtained from the patient, caregiver, or a legal representative; (4) discharged home; (5) mild stroke operationalized as mild disability at discharge as defined by a modified Rankin scale score of 1 to 2; and (6) ambulatory at the time of hospital discharge. The modified Rankin scale is a 1-item clinician-reported scale with scores ranging from 0 (representing no symptoms) to 6 (death) [22]. Patients with modified Rankin scale scores of 1 to 2 have residual symptoms but are able to ambulate without assistance from another person. Exclusion criteria were the following: (1) aged <18 years; (2) prisoners; (3) ischemic stroke due to vasculitis, moya-moya, complication from surgical procedure, or trauma; (4) non-English speaking patient with no available proxy; (5) residing in hospice or receiving palliative care prior to enrollment; (6) active recreational drug use; and (7) a medical condition that would impair the patient's ability to participate in this study in the opinion of the investigator.

Most patients discharged home after stroke from the inpatient stroke service had few neurological deficits as assessed by the clinician-reported National Institutes of Health Stroke Scale [23], a commonly used scale of severity of deficits in patients with stroke. To include participants with slightly greater deficits, the inclusion criteria were modified in the final 2 months of the recruitment period to include stroke patients discharged from inpatient rehabilitation units.

**Figure 1:**
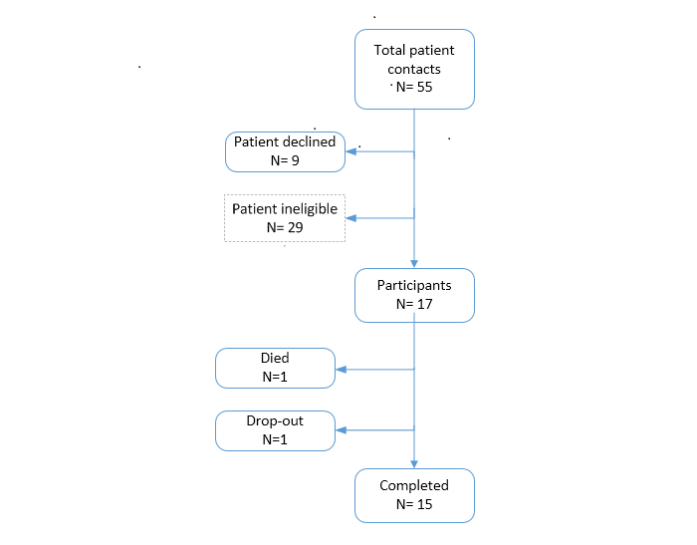
Study enrollment and retention.

A total of 55 individuals were approached to participate in the study. Of these, 11 declined participation and 29 no longer met the inclusion criteria after initial interest in participating (Figure 1). Reasons these patients failed to meet inclusion criteria were (1) not having an internet-facing electronic device that could link to the Fitbit and upload step count data through the internet (n=10); (2) nonstroke final diagnosis (n=8); (3) poor medical compliance (n=3); (4) cognitive deficits (n=4); (5) unavailable prior to hospital discharge (after first contact) (n=2); and (6) already had Fitbit account on their personal device precluding synchronization of their device with a research account required for study participation (n=2). A total of 17 patients were enrolled in the study; one participant died due to factors unrelated to this study approximately 2 weeks postdischarge, and another participant dropped out of the study shortly after completing the assessment at hospital discharge because she felt the Fitbit Charge HR was uncomfortable on her wrist. These 2 patients were not included in the analyses.

#### Step Activity Monitor Data

The Fitbit Charge HR was provided to patients at the time of enrollment. It is a wrist-worn device with a triaxial accelerometer marketed as a consumer device that displays step count and wirelessly synchronizes with smartphones. Fitbit devices have been shown to provide step counts similar to those provided by research grade accelerometers [19,20], including in patients with stroke [15]. MyInertia platform (Motion Connected LLC, Green Bay, WI) was used for administrative access to the Fitbit total daily step count tallies of study participants. The MyInertia platform did not provide a more precise timing of steps within a 24-hour period, so we were unable to determine when patients ambulated or wore the Fitbit within a 24 hour period. A MyInertia account linked to each patient’s Fitbit device was activated at the beginning of the study and deactivated at the end of the study period.

#### Mobility Performance Tests

We administered 3 mobility performance tests that are part of the National Institutes of Health (NIH)Toolbox at discharge and at a return visit on Day 30: (1) Standing Balance Test, (2) 2-Minute Walk Endurance Test, and (3) 4-Meter Walk Gait Speed Test. The NIH Toolbox is comprised of numerous standardized royalty-free measures in neurological and behavioral health. It is designed for use in people ages 3 to 85 to allow for comparison of data across multiple studies [24]. The tests are administered through a downloaded application onto an iPad and each provides automated scoring. Balance has been shown to be independently associated with step counts [25], and the 2-Minute Walk Endurance Test and 4-Meter Walk Gait Speed Test are standard performance tests in stroke. The National Institutes of Neurological Disorders and Stroke (NINDS) Stroke Clinical Data Elements (CDE) Project considers walking speed a “highly recommended” outcome measure in patients with stroke [26].

The NIH Toolbox balance test involves the participant maintaining 5 poses for 50 seconds each. Postural sway is recorded for each pose using an accelerometer worn by the participant which synchronizes with the NIH Toolbox app. Normative scores are provided that adjust for age, sex, ethnicity, and education. The average score of the normative population is 50 (SD 10), with higher scores indicating better balance.

The 4-Meter Gait Speed Test measures the time required to walk 4 meters, which is then transformed into gait speed in meters per second. The 2-Minute Walk Endurance Test records the distance that the participant is able to walk back and forth on a 50-foot course in 2 minutes, which is converted to normative scale scores. As with balance scores, higher scores indicate better performance. To determine accuracy of the step activity monitor, we manually counted the number of steps during the 2-Minute Walk Endurance Test using a hand counter at both discharge and Day 30 assessments.

#### Clinician-Reported Measures

The National Institutes of Health Stroke Scale [23] is the standard scale for measuring neurological impairment. It consists of 15 items with scores ranging from 0 to 42, with higher scales indicating greater impairment. The modified Rankin scale is a 1-item measure of global disability, with scores ranging from 0 to 6 where 0 represents no symptoms [22]. These measures were part of the study inclusion criteria and were used to describe the study participants.

#### Participant Experience Survey (Secondary Objective 4)

Questions were adapted from those used in previously published research [27-30] with the addition of new questions specific to our study (See Multimedia Appendix 2: Participant Experience Survey).

### Statistical analysis

#### Primary Objective: Adherence With Wearing a Step Activity Monitor

The MyInertia system used to access step count data for this study only provided daily step counts. Participant adherence was defined as having a daily step count of 100 or more. This threshold was chosen to minimize the chance of incorrectly classifying participants as nonadherent. Available data from the literature indicates that stroke survivors are sedentary [4,6]. Our study participants were recently discharged home after a hospital admission for stroke, and it was possible they would not be very physically active, especially in the first few days after discharge.

Adherence was summarized descriptively and modeled using a logistic Generalized Estimating Equation. A first-order auto-regressive (AR(1)) correlation structure was used to take into account the dependent nature of consecutive days. The intercept of the Generalized Estimating Equation model provided the population-level probability of adherence. This approach to calculating adherence allowed more accurate estimates of error around adherence rate [31].

#### Secondary Objectives

##### Secondary Objective 1: Determine Fitbit Accuracy

To determine accuracy of the Fitbit, we performed a manual count of the number of steps taken during the 2-Minute Walk Endurance Test using a hand tally counter at both discharge and Day 30 assessments. We performed an analysis of differences in the number of steps recorded on the Fitbit during 2-Minute Walk Endurance Tests and manual tally by calculating mean and median differences and mean absolute difference. Differences between device and manually counted steps were graphically displayed using Bland-Altman plots for repeated measures [32]. Accuracy of step counts was considered “acceptable” if variance between the manual tally and Fitbit step count was within 10% in either direction [33-35].

##### Secondary Objective 2: Calculate Correlation Between Step Counts and Mobility Performance Scores

Spearman rank correlations were used to examine the relationship between step counts and mobility performance test scores for the weeks following discharge and the Day 30 visit. The mean daily steps of the 7-day period following assessments was used to summarize step counts. Confidence intervals were estimated using the bootstrap with 10,000 repetitions. As per standard NIH Toolbox scoring procedures, these mobility performance tests were adjusted for age, sex, and education to provide normative data for patients falling within similar age, sex, and educational attainment categories [36].

##### Secondary Objective 3: Determine Variability in Step Counts

Variability in steps-per-day was measured over the 90-day study period and for each 7-day period. Previous studies have found that a 7-day monitoring period is required to obtain a stable and representative average of levels of walking activity in healthy people [37] and those with neurological impairment [38]. Variability was assessed using a mixed effects model with a subject-level random intercept. Between-subjects variability was estimated using the subject-level random effect variance. Within-subject variability was indirectly assessed by calculating the intraclass correlation defined here as the proportion of total variance explained by between-subjects variability; the higher the intraclass correlation coefficient value, the greater the between-subjects variability.

##### Secondary Objective 4: Evaluate Participant Experience

Descriptive statistics were used to summarize participant survey responses.

#### Sensitivity Analyses for Definition of Daily Adherence

The suitability of the definition of daily adherence used in our analysis was evaluated using self-reported daily diaries during the first 7 days of monitoring. Daily diary entries were considered adherent if a patient reported wearing the device for more than 12 hours per day. Diary entries indicating that the Fitbit was not worn for 12 or more hours were categorized as nonadherent. Agreement between the diaries and adherence was summarized as a percentage. In a sensitivity analysis, the percentage of participants identified as “adherent” was calculated for different daily step count thresholds.

In addition, a sensitivity analysis was conducted to evaluate the effect of varying adherence thresholds on correlations between mobility performance scores and step counts. This was done by computing a series of average daily step counts over the 7-day period immediately following the performance measure assessment using only the days where the total step count was at or above specific thresholds (no step count restriction, ≥100, ≥500, ≥1000, ≥2000, ≥3000, ≥4000, and ≥5000 steps). Correlations between the mobility performance scores and each series of daily step counts was then calculated.

#### Sample Size Calculations

Sample size calculations were based on simulations to determine power required for the primary objective. We assumed that the percentage of adherent days could vary from 50% to 96% [37,39-41] with an average of 76%. With a sample size of 15 participants and 90 days of data from each participant, the estimated power was greater than 73% to detect a 1% or greater difference above the null hypothesis adherence rate of 75% [37,41].

Power for secondary objective 2, the correlation between mobility performance scores and step counts, was estimated using 1000 simulations of bivariate normal distributed data with correlations of 0.65. Power for a sample size of 15 was estimated to be greater than 60% to detect a correlation greater than zero.

All computations were done in R, version 3.4.1 [42]. All tests were two-sided and *P* values less than .05 were considered statistically significant.

## Results

### Characteristics of Participants

Participants had a mean age of 54.4 years (SD 12.1, range 34-81 years) and 60% (9/15) were male. Most had at least some college education (11/15, 73.3%). Participants were 60% (9/15) non-Hispanic White. Discharge National Institutes of Health Stroke Scale score was 0 or 1 in 73% (11/15) of participants (Table 1). Of the 15 participants, 3 (20%) indicated they used a cane at the Day 30 visit, although no ambulatory assistance devices were used during performance measure testing. The number of mean daily steps during the first week of monitoring was 4368 (SD 3968), with individual participants means ranging from 1140 to 14610. The majority of participants (12/15, 80%) could be classified as sedentary (ie, mean daily step count under 5000 [43]).

Scores for the Standing Balance Test, 2-Minute Walk Endurance Test, and 4-Meter Gait Speed Test measured at discharge and Day 30 are presented in Table 2. Fully-corrected T-scores were adjusted for age, education, race, and sex, and have a mean of 50 (SD 10) in the normative population.

**Table 1:** Participant characteristics (N=15).

Variable		Value
**Age, years**		
	Mean (SD)	54.4 (12.1)
	Range	34-81
**NIHSS^a^, n (%)**		
	0	6 (40)
	1	5 (33.3)
	3	3 (20)
	4	1 (6.7)
Male, n (%)		9 (60)
White Non-Hispanic, n (%)		9 (60)
**Marital status, n (%)**		
	Married	9 (60)
	Divorced	1 (6.7)
	Widowed	1 (6.7)
	Single	4 (26.7)
**Discharge Modified Rankin Scale, n (%)**		
	0	2 (13.3)
	1	8 (53.3)
	2	5 (33.3)
Received PT^b^ and/or OT^c^ post-discharge, n (%)		9 (60)
Use of ambulatory assistance device^d^, n (%)		3 (20)
**Education, n (%)**		
	High School	4 (26.7)
	Some college	6 (40)
	Associate's degree	3 (20)
	Bachelor’s degree	1 (6.7)
	Master's degree	1 (6.7)
Right-handed, n (%)		15 (100)

^a^NIHSS: National Institutes of Health Stroke Scale.

^b^PT: physical therapy.

^c^OT: occupational therapy.

^d^Used a cane at Day 30 visit.

**Table 2:** Mobility performance tests at Discharge and Day 30. Scores adjusted for age, sex, race/ethnicity, and education. Average T-Score is 50; higher scores indicate better performance. 2-Minute Walk Test is a measure of endurance; 4-Meter Distance Test is a measure of gait speed.

Measure	Discharge		Day 30 follow-up		Difference		
	Mean (SD)	Range	Mean (SD)	Range	Mean (SD)	Range	*P* value^a^
2-minute walk T-Score	27.94 (9.78)	14.52 to 45.16	33.53 (8.09)	19.39 to 48.75	5.60 (7.38)	–3.52 to 19.71	.01
Balance T-Score	40.75 (13.67)	24.47 to 64.26	40.55 (12.98)	22.96 to 63.68	0.64 (10.85)	–19.6 to 17.4	.83
4-meter distance T-Score	43.2 (13.46)	24.95 to 61.94	50.15 (15.23)	27.68 to 72.67	6.95 (11.86)	–16.34 to 28.34	.04
2-minute distance (m)	105.43 (40.9)	31.9 to 164.59	130.92 (33.08)	79.25 to 180.98	25.49 (29.31)	–20.42 to 83.82	.004
4-meter speed (m/sec)	0.94 (0.35)	0.2 to 1.43	1.16 (0.36)	0.67 to 1.79	0.22 (0.28)	–0.24 to 0.75	.01

^a^*P* value from paired-samples *t* test with unequal variance.

On average, participants performed worse than the general population after adjustment for education, sex, and age on all 3 tests, especially on the 2-Minute Walk test. Participant scores for both mobility tests were significantly improved at the Day 30 follow-up; balance test scores were similar at both time points.

### Primary Objective: Adherence With Wearing the Step Activity Monitor

Using the adherence definition of ≥100 steps, participants were adherent 83.6% of days (approximately n=75) during the 90 day study period. An intercept-only logistic Generalized Estimating Equation using an AR(1) correlation, which adjusts for the correlation of adjacent daily observations, estimated the probability of adherence as 0.83 (95% CI 0.73-0.90). Three participants with low-adherence reported difficulties with the device: the wristband broke, step activity monitor was lost when traveling, the charger was lost. We replaced 2 of these devices. Removing these days from the calculations, probability of adherence was 0.86 (95% CI 0.78-0.92).

### Secondary Aims

#### Secondary Objective 1 : Determine Fitbit Accuracy

**Figure 2:**
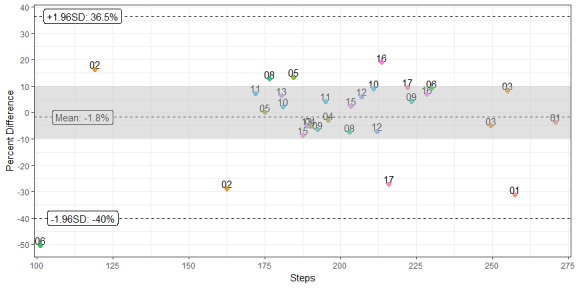
Bland Altman plot of accuracy of step activity monitor step counts compared to manual counts. Percent difference between Fitbit and manual step counts (y-axis) versus the manual step counts (x-axis). Each participant had 2 trials. The shaded region represents the “acceptable” range of error (10% in either direction), while dotted lines depict the mean percent difference and their 95% confidence intervals. Participants 2, 6, and 8 used a cane for ambulation, but no ambulatory assistance devices were used during gait testing.

While individual observation step counts recorded by the Fitbit displayed variability in comparison to manual tallies, the overall mean difference was only –4.8 steps (–1.8%). Mean absolute difference was higher, at approximately 21.7 steps (10.9%). A Bland-Altman plot showing percent difference between Fitbit and manual counts versus manual count appears in Figure 2. Observations were within an acceptable margin of error, defined as a 10% difference from manual counts, 73.3% of the time.

#### Secondary Objective 2: Calculate Correlations Between Step Counts and Mobility Performance Scores

Correlations between step counts and mobility performance scores at both time points were very poor. Values for 95% confidence intervals all straddled zero. Correlations with endurance (2-Minute Walk Endurance Test v2.0), balance (Standing Balance Test v2.0), and gait (4-Meter Walk Gait Speed Test v2.0) at discharge were 0.44 (95% CI –0.14 to 0.75), –0.12 (95% CI –0.67 to 0.64), and 0.17 (95% CI –0.46 to 0.66), respectively. Correlations at Day 30 were 0.22 (95% CI –0.45 to 0.71), –0.30 (95% CI –0.83 to 0.41), and 0.21 (95% CI –0.48 to 0.76) respectively.

#### Secondary Objective 3: Determine Variability in Step Counts

Intraclass correlation showed moderate within-subject correlation (intraclass correlation coefficient=0.47) in step counts. Table 3 depicts intraclass correlation and between-subject variability (expressed as standard deviation) for 7 day periods over the course of the study. Values suggest moderate to large between-subject and within-subject variability. As the study progressed, between-subject variability decreased while within-subject variability increased, as shown by lower intraclass correlations.

**Table 3:** Variability in weekly step counts.

Week	Within-subject variability, intraclass correlation coefficient (95% CI)^a^	Between-subject variability, standard deviation (95% CI)^b^
1	0.73 (0.49-0.84)	3338.02 (1997.56-5218.09)
2	0.48 (0.18-0.66)	2367.04 (1539.35-3639.75)
3	0.39 (0.10-0.60)	2089.70 (1294.69-3372.90)
4	0.68 (0.40-0.81)	2967.29 (2011.54-4377.15)
5	0.40 (0.13-0.61)	2016.81 (1281.03-3175.19)
6	0.54 (0.26-0.70)	2504.30 (1665.16-3766.31)
7	0.34 (0.09-0.53)	1859.55 (1143.88-3022.99)
8	0.53 (0.21-0.71)	2222.68 (1435.35-3441.88)
9	0.41 (0.10-0.61)	2040.17 (1242.61-3349.64)
10	0.48 (0.18-0.66)	2252.09 (1445.39-3509.02)
11	0.45 (0.14-0.65)	2196.83 (1354.26-3563.62)
12	0.36 (0.07-0.57)	1978.23 (1185.71-3300.49)
13^c^	0.39 (0.04-0.62)	1639.84 (972.28-2765.745

^a^95% confidence interval estimated using semiparametric bootstrap with 10,000 resamples.

^b^95% confidence interval calculated using the Wald-type test.

^c^6-day period from day 85 to 90. Over time, between-subject variability decreased while within-subject variability increased, as shown by lower intra-class correlations.

#### Secondary Objective 4: Evaluate Participant Experience

**Figure 3:**
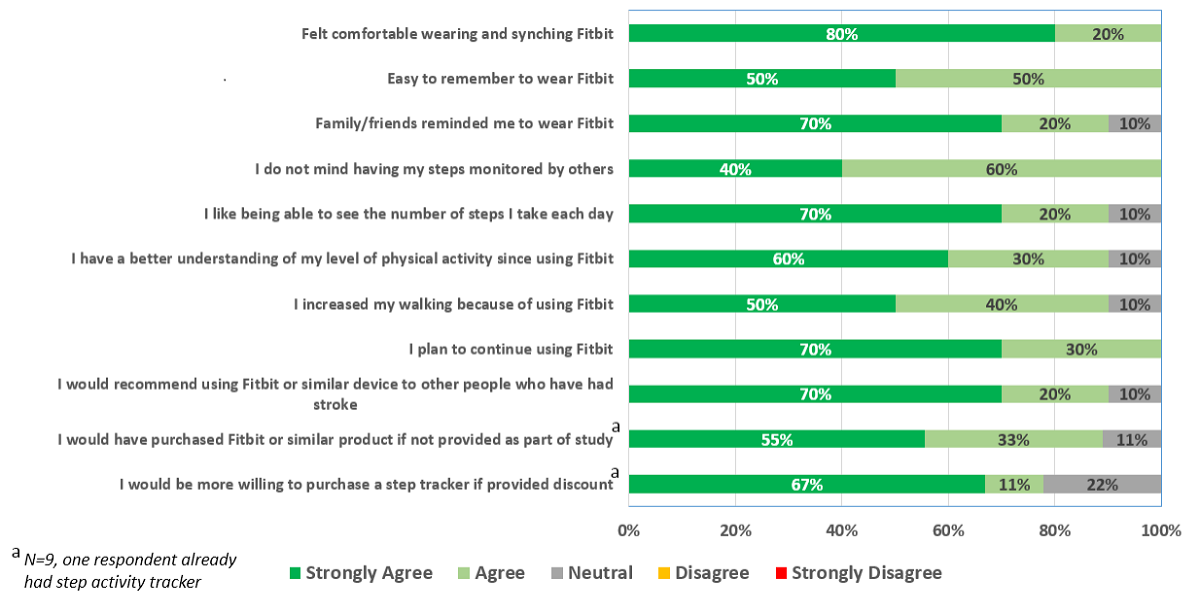
Participant experience survey responses (n=10).

Of the 15 participants, 10 (67%) completed the participant experience survey. All felt comfortable wearing the Fitbit device, thought it was easy to remember to wear, and planned to continue to use it (Figure 3). None of the respondents minded having their step count activity monitored by others. The majority (9/10, 90%) felt they had a better understanding of their physical activity levels and would recommend a step activity monitor to other people that have had a stroke.

### Sensitivity Analyses for Definition of Daily Adherence

The suitability of the definition of daily adherence used in our analysis was evaluated using self-reported daily diaries during the first 7 days of monitoring**.** Of the 15 participants, 8 (53.3%) filled out daily diaries of physical activity for the first 7-day period that they wore their Fitbit. These participants reported complete adherence (>12 hours/day Fitbit use) for that 1-week time period. During that 1-week period, step counts for these 8 participants were over 100 steps per day; thus, there was 100% agreement between the diary and step counts using definition of adherence of 100 or more steps. The minimum daily tally during the 7-day period was 346 steps. The percentage agreement for adherence decreased to 96.2% when using 1000 steps as a threshold, as 2 of the 8 participants (25%) had a single daily step count entry with less than 1000 steps.

An additional sensitivity analysis was performed to evaluate if the use of different thresholds affected the correlations between mobility performance scores and daily step counts. Altering the thresholds did not improve correlations consistently between the different mobility performance measure scores and average 7-day step count (See Multimedia Appendix 3: Spearman rank correlations between performance evaluations and different daily step count thresholds).

## Discussion

### Principal Findings

Our study demonstrated that wearing a consumer-grade step activity monitor after stroke is feasible in patients with recent mild acute ischemic stroke. Participants were adherent 83.6% of days over the 90-day study period, which included several instances of device malfunction or misplacement. We defined adherence a priori as ≥100 steps per day, although varying the step-counts thresholds had little effect either on the percentage of days participants were labeled adherent or with the correlation between step counts and participant performance on mobility tests. Studies of adherence with step activity monitors are sparse, and the differences in definition of adherence, populations studied, and duration of monitoring make comparisons across studies difficult. A recent clinical trial of step activity monitor use in patients with rheumatoid arthritis [44], which monitored step counts over a 21-week period, demonstrated an adherence rate of 88.8% per study day, similar to our study. That study defined adherence as the proportion of study days that steps were recorded.

The accuracy of step activity monitors is a critical consideration when choosing a device, especially in patients with neurological impairment. This study suggests that the Fitbit Charge HR provides reasonably accurate step counts in patients with mild stroke who have similar gait speeds to those in our study. While observed step counts of individuals displayed high variability in accuracy, overall mean error was only –4.8 steps (–2.4%). The mean gait speed of participants in our study was 0.94 m/sec and the accuracy of the Fitbit Charge HR in patients with slower gait speeds than observed in our study is unclear [45].

Approximately 58% (15/26) of eligible patients completed the study. Almost 20% of patients approached to participate (10/55) were ineligible because they did not have an internet-facing electronic device that could link to the Fitbit, an important factor to consider when planning future studies of step counts in stroke. This rate is likely to decline with time but could be a significant factor limiting recruitment over the next few years. Acceptability of wearing the Fitbit was quite high. The majority of participants who responded to the survey indicated they would continue to use the devices to monitor their physical activity after the study ended. The respondents also indicated they would not mind having this information be available to providers.

There are several advantages to using consumer-grade step activity monitors to monitor ambulation in patients with stroke and other conditions. Designed for the consumer, they may be more intuitive for patients to use, provide user-friendly displays, and often collect other data such as sleep time and heart rate. These factors may have contributed to the high adherence rates and acceptability of the device in our study. Healthcare wearables, such as step activity monitors, have been predicted to grow from 1 million purchases in 2015 to 97.6 million by 2021 [46]. More stroke patients will already own and wear their own step activity monitor in the coming years, another significant advantage. In addition, several electronic health record companies are developing conduits to pull in patient-generated heath data from commonly used consumer devices making them easier to incorporate into providers’ clinical care workflows.

Although this study did not directly assess the clinical utility of step activity monitors for patients with mild stroke, the results provide some useful insights for the design of future studies that address this question. First, most of the participants in our study were sedentary, walking less than 5000 steps per day. Our findings are consistent with others who found that many stroke survivors have low levels of physical activity [4,6] and highlights the merit of research regarding the effectiveness of step targets in increasing the ambulation of persons with stroke. Second, there was wide variability in weekly step counts both within and across study participants, which has implications for determining sample sizes of future studies that aim to identify trends. A significant trade-off between sensitivity and specificity to detect change may be required when using step activity monitors in stroke survivors.

A notable and somewhat unexpected finding in our study was the dismal correlation between step counts and performance measures for gait speed, endurance, and balance. The endurance and gait speed tests are standard performance tests in stroke [26] and, along with the balance test, have been shown to be independently associated with step counts [5,6,25,47]. Testing was performed using tools in the NIH Toolbox, which are validated and recommended for use in clinical trials to allow for standard assessment. The low correlations highlight important distinctions between step counts and mobility performance measures that, in light of these findings, may be especially pertinent in patients recovering from mild stroke. Performance measures assess what a patient is capable of doing in ideal conditions, whereas step count measures activity in real-world conditions. Patients’ habits, social environment, and attitudes towards engaging in physical activities affect the number of steps taken apart from their physical abilities. Low mood, for example, can contribute to lower physical activity levels [6]. It is possible that there is a stronger association between step counts and patient-reported outcomes, which are also impacted by patient environment and attitudes. It will be important to assess the relationship between step counts in stroke patients and patient-reported and other outcome measures to provide clinical relevance and interpretation of step count values.

### Strengths and Limitations

An important strength of our study included the 90-day monitoring period. A 7-day protocol for step activity monitors is commonly used in research protocols [48-51]. Assessment of adherence over 90 days provides information on the feasibility of using step activity monitors during the initial 3 month period after stroke (the timeframe in which the most rapid recovery occurs [52]) and for studies of behavioral intervention that may last months rather than weeks. Another strength is the collection of performance measures across 2 time points, which allowed assessment of whether correlations between step count and mobility performance tests varied according to the time since stroke.

This study also has several limitations. Patient participants had overall mild degree of disability and findings from our study may not be generalizable to stroke survivors with more severe deficits. Our initial target population consisted of patients discharged home after stroke admission, since ambulation in the acute rehabilitation setting can be constricted [53]. We modified the protocol to include patients discharged from acute rehabilitation to assess step counts in patients with greater deficits. The feasibility study included 15 participants and findings would be more compelling with a larger sample size. In addition, only two-thirds (10/15) of the participants completed the participant experience survey. Another important limitation is that we were unable to evaluate within-day variability, intensity, and patterns of step counts. A recent study of patients with Parkinson’s disease [54] found that intensity of physical activity, but not step counts, declined over time, suggesting that intensity or type of activity could be more sensitive to detect change in physical function. The lack of detailed step count activity within a 24-hour period also constrained our assessment of adherence. However, benefits of using daily step counts include their measurement simplicity and higher likelihood of being available within a patient’s electronic health record. Identifying when a participant is not wearing the step activity monitor is difficult even when detailed step activity data is available within a 24-hour period [55]. An important avenue of further research is to determine the optimal definition of adherence when only daily step count totals are available. One approach may be individualization of step count threshold for each person based on their baseline step counts and activity patterns.

There are also drawbacks with using consumer-grade devices in general. The algorithms used to measure steps and other metrics are typically proprietary and may not be available to investigators [56]. Consumer-grade devices such as the Fitbit Charge HR are often worn on the wrist, and these are not suitable for monitoring step counts in patients using rollator ambulation aids [15].

### Conclusions

The use of patient-generated health data in clinical care and research is part of an evolving paradigm shift in health care. Wearable technologies such as step activity monitors offer an excellent mechanism for gathering data from patients effectively, continuously, and in real time. This evaluation represents one of the first applications of patient-generated health data from consumer wearable devices in patients with stroke. The results of this study suggest that the use of the consumer-grade Fitbit Charge HR in patients with recent mild stroke is feasible with reasonable adherence, accuracy, and high level of acceptability. There was poor correlation between step counts and gait speed, balance, and endurance as assessed using the mobility performance tests in the NIH Toolbox. Further research is needed to evaluate the association between step counts and other outcomes of relevance to patients, including patient-reported outcomes and measures of physical function. This feasibility study will serve as important groundwork for further studies of the use of step activity monitors to optimize the care and outcomes of patients with stroke.

## Appendix

Multimedia Appendix 1 Participant activity diary.

Multimedia Appendix 2 Patient experience survey.

Multimedia Appendix 3 Spearman rank correlations between performance evaluations and different daily step count thresholds.

## Abbreviations

NIH: National Institutes of Health
