# Supplementary material for: Physical Activity Monitoring Using a Fitbit Device in Ischemic Stroke Patients: Prospective Cohort Feasibility Study
Source: JMIR Mhealth Uhealth. 2021 Jan 19;9(1):e14494. doi: 10.2196/14494 (PMC7854036; doi:10.2196/14494)
Supplement: Multimedia Appendix 1 [file mhealth_v9i1e14494_app1.docx]

**Multimedia Appendix 1 – Participant Activity Diary**

Day 1 – *Example for Participant to follow*

Date: Thursday, September 1, 2016

| **Time** | **Wear Fitbit** | **Describe physical activity (awake/sleep, activities outside of sitting, walking within the house)** |
| --- | --- | --- |
| 6:00am | x | *awake* |
| 7:00am |  | *shower* |
| 8:00am | X |  |
| 9:00am | X | *Physical therapy session* |
| 10:00am | X |  |
| 11:00am | X | *Mowed lawn* |
| 12:00am | X |  |
| 1:00pm | X |  |
| 2:00pm | X | *1 hour nap* |
| 3:00pm | X |  |
| 4:00pm |  | *swimming* |
| 5:00pm | X |  |
| 6:00pm | X |  |
| 7:00pm | X | *Walked dog* |
| 8:00pm | X |  |
| 9:00pm | X |  |
| 10:00pm | X | *sleep* |
| 11:00pm | X |  |
| 12:00pm | X |  |
| 1:00am | X |  |
| 2:00am | X |  |
| 3:00am | X |  |
| 4:00am | X |  |
| 5:00am | X |  |

Notes:

Physical Activity Diary

Day 1

Date:

| **Time** | **Wear Fitbit** | **Describe physical activity** |
| --- | --- | --- |
| 6:00am |  |  |
| 7:00am |  |  |
| 8:00am |  |  |
| 9:00am |  |  |
| 10:00am |  |  |
| 11:00am |  |  |
| 12:00am |  |  |
| 1:00pm |  |  |
| 2:00pm |  |  |
| 3:00pm |  |  |
| 4:00pm |  |  |
| 5:00pm |  |  |
| 6:00pm |  |  |
| 7:00pm |  |  |
| 8:00pm |  |  |
| 9:00pm |  |  |
| 10:00pm |  |  |
| 11:00pm |  |  |
| 12:00pm |  |  |
| 1:00am |  |  |
| 2:00am |  |  |
| 3:00am |  |  |
| 4:00am |  |  |
| 5:00am |  |  |

Notes:
