# Supplementary material for: Physical Activity Monitoring Using a Fitbit Device in Ischemic Stroke Patients: Prospective Cohort Feasibility Study
Source: JMIR Mhealth Uhealth. 2021 Jan 19;9(1):e14494. doi: 10.2196/14494 (PMC7854036; doi:10.2196/14494)
Supplement: Multimedia Appendix 2 [file mhealth_v9i1e14494_app2.docx]

| **Multimedia Appendix 2. Patient Experience Survey** | | | | | | | | | | |  |
| --- | --- | --- | --- | --- | --- | --- | --- | --- | --- | --- | --- |
|  | |  | | ***Please clearly mark the most appropriate response:*** | | | | | | |  |
| 1 | | I had a Fitbit or other step activity monitor prior to my stroke | | **YES** | | **NO** |  | |  |  | |
|  | |  | | **Strongly agree** | | **Agree** | **Neutral** | **Disagree** | | | **Strongly disagree** |
| 2 | | I felt comfortable using the Fitbit and syncing it to my smartphone, tablet or computer | |  | | **** | **** | **** | | | **** |
| 3 | | It was easy to remember to **wear** the Fitbit | | **** | | **** | **** | **** | | | **** |
| 4 | | It was easy to remember to **charge** the Fitbit | | **** | | **** | **** | **** | | | **** |
| 5 | | Family and/or friend(s) encouraged and/or reminded me to wear my Fitbit | | **** | | **** | **** | **** | | | **** |
| 6 | | I do not mind having my steps and activity monitored by others | | **** | | **** | **** | **** | | | **** |
| 7 | | I like being able to see the number of steps I take each day | | **** | | **** | **** | **** | | | **** |
| 8 | | I have a better understanding of my level of physical activity since using the Fitbit | | **** | | **** | **** | **** | | | **** |
| 9 | | I increased my walking or other physical activity because of using the Fitbit | | **** | | **** | **** | **** | | | **** |
| 10 | | I plan to continue using the Fitbit device after the study ends | | **** | | **** | **** | **** | | | **** |
| 11 | | I would recommend using a Fitbit or similar device to other people that have had a stroke | | **** | | **** | **** | **** | | | **** |

**Patient Experience Survey - continued**

|  |  | **Strongly agree** | **Agree** | **Neutral** | **Disagree** | **Strongly disagree** |
| --- | --- | --- | --- | --- | --- | --- |
| 12 | I would have purchased (spent my own money) on a product like a Fitbit if it was not provided as part of the study | **** | **** | **** | **** | **** |
| 13 | I would be more willing to purchase a Fitbit or similar device if I was incentivized (was offered a discount on cost of the device or on health insurance premium) | **** | **** | **** | **** | **** |
| 14 | Would you be open to being contacted again in the future regarding this study after your participation has ended? | **YES** | **NO** |  |  |  |
| 15 | I am satisfied with the amount of communication I received from the study coordinator | **** | **** | **** | **** | **** |
| 16 | Do you have any suggestions for improvement for our study? | ***provide free text comments here:*** | | | | |
