# Supplementary material for: Physical Activity Monitoring Using a Fitbit Device in Ischemic Stroke Patients: Prospective Cohort Feasibility Study
Source: JMIR Mhealth Uhealth. 2021 Jan 19;9(1):e14494. doi: 10.2196/14494 (PMC7854036; doi:10.2196/14494)
Supplement: Multimedia Appendix 3 [file mhealth_v9i1e14494_app3.docx]

**Multimedia Appendix 3. Spearman rank correlations between performance evaluations and different daily step count thresholds**.

| **Daily Steps^a^** | **Discharge** | | | | | **Day 30** | | | | |
| --- | --- | --- | --- | --- | --- | --- | --- | --- | --- | --- |
|  | **2-min Walk Endurance Test T-score (95% CI)** | **Balance Test**  **T-score**  **(95% CI)** | **4-meter Gait Speed Test**  **T-score**  **(95% CI)** | **2-min Walk Distance (m)**  **(95% CI)** | **Gait Speed (m/sec)**  **(95% CI)** | **2-min Walk Endurance**  **T-score**  **(95% CI)** | **Balance Test**  **T-score**  **(95% CI)** | **4-meter Gait Speed Test**  **T-score**  **(95% CI)** | **2-min Walk Distance (m)**  **(95% CI)** | **Gait Speed (m/sec)**  **(95% CI)** |
| **No Restriction** | 0.41  (-0.15, 0.75) | -0.12  (-0.67, 0.64) | 0.17  (-0.47, 0.67) | 0.39  (-0.09, 0.7) | 0.19  (-0.47, 0.67) | 0.17  (-0.48, 0.66) | -0.11  (-0.75, 0.61) | 0.05  (-0.56, 0.6) | 0.13  (-0.53, 0.65) | 0.1  (-0.5, 0.61) |
| **≥100** | 0.41  (-0.14, 0.75) | -0.12  (-0.67, 0.64) | 0.17  (-0.46, 0.66) | 0.39  (-0.09, 0.7) | 0.19  (-0.46, 0.67) | 0.22  (-0.45, 0.71) | -0.3  (-0.83, 0.41) | 0.21  (-0.48, 0.76) | 0.21  (-0.49, 0.77) | 0.27  (-0.41, 0.77) |
| **≥500** | 0.36  (-0.24, 0.72) | -0.09  (-0.65, 0.64) | 0.18  (-0.46, 0.62) | 0.39  (-0.14, 0.72) | 0.21  (-0.4, 0.63) | 0.22  (-0.45, 0.72) | -0.3  (-0.8, 0.44) | 0.21  (-0.47, 0.76) | 0.21  (-0.49, 0.77) | 0.27  (-0.39, 0.78) |
| **≥1,000** | 0.53  (0.05, 0.78) | 0.02  (-0.64, 0.7) | 0.27  (-0.42, 0.71) | 0.57  (0.14, 0.82) | 0.3  -0.34, 0.72) | 0.2  (-0.46, 0.68) | -0.29  (-0.79, 0.49) | 0.19  (-0.52, 0.74) | 0.19  (-0.5, 0.76) | 0.24  (-0.43, 0.75) |
| **≥2,000** | 0.55  (0.02, 0.85) | -0.07  (-0.81, 0.76) | 0.19  (-0.54, 0.74) | 0.47  (-0.15, 0.8) | 0.16  (-0.58, 0.75) | 0.17  (-0.46, 0.69) | -0.22  (-0.77, 0.52) | 0.19  (-0.51, 0.74) | 0.18  (-0.52, 0.75) | 0.25  (-0.41, 0.76) |
| **≥3,000** | 0.48  (-0.14, 0.83) | -0.02  (-0.8, 0.8) | 0.38  (-0.47, 0.84) | 0.42  (-0.31, 0.87) | 0.38  (-0.43, 0.85) | -0.05  (-0.61, 0.54) | -0.21  (-0.77, 0.55) | 0.06  (-0.61, 0.7) | -0.06  (-0.67, 0.6) | 0.17  (-0.54, 0.77) |
| **≥4,000** | 0.47  (-0.44, 0.89) | -0.37  (-0.93, 0.73) | 0.35  (-0.69, 0.89) | 0.3  (-0.66, 0.85) | 0.35  (-0.67, 0.89) | -0.17  (-0.8, 0.54) | -0.07  -0.73, 0.68) | 0.2  (-0.62, 0.82) | -0.1  (-0.76, 0.63) | 0.38  (-0.43, 0.84) |
| **≥5,000** | 0.02  (-0.89, 0.76) | -0.9  (-1, -0.41) | 0.08  (-0.8, 0.82) | -0.2  (-0.89, 0.71) | 0.08  (-0.8, 0.84) | -0.19  (-0.82, 0.57) | -0.08  (-0.79, 0.64) | 0.16  (-0.65, 0.85) | -0.12  (-0.79, 0.67) | 0.35  (-0.52, 0.84) |

CI = confidence intervals. 95% CI were estimated using the adjusted bootstrap percentile method with 10,000 iterations. ^a^Average daily step count over the 7-day period following assessment.
